# Severe neuropsychiatric manifestations in a 17-year-old male with a 20p13 interstitial deletion encompassing *CSNK2A1*: a case report

**DOI:** 10.1186/s13256-026-06800-7

**Published:** 2026-11-02

**Authors:** Joern Pascal Laubach, Daria Zeeb, Stefanie Weber, Katja Becker, Peter Seipelt

**Affiliations:** 1 Department of Child and Adolescent Psychiatry, Psychosomatics and Psychotherapy, University of Marburg, Marburg, Germany; 2 Department of Pediatrics, GFO-Kliniken Bonn, St. Marienhospital Bonn, Robert-Koch-Straße 1, 53115 Bonn, Germany; 3 Center for Human Genetics Marburg, University of Marburg, Marburg, Germany; 4 Department of Pediatrics, University of Marburg, Marburg, Germany; 5 Pediatric Neurology, Department of Pediatrics, University of Marburg, Marburg, Germany

**Keywords:** 20p13 microdeletion, *CSNK2A1* gene, Okur-Chung neurodevelopmental syndrome, Neurodevelopmental disorder, Intellectual disability, Microcephaly, Case report

## Abstract

**Background:**

Microdeletions of 20p13 involving *CSNK2A1* have rarely been reported and may be associated with an Okur-Chung neurodevelopmental syndrome compatible phenotype. Okur-Chung neurodevelopmental syndrome is a rare neurodevelopmental syndrome (OCNDS) caused by heterozygous pathogenic variants in *CSNK2A1* and is characterized by a broad spectrum of symptoms including developmental delays and intellectual disability.

**Case presentation:**

This case report describes a 17-year-old male with de novo interstitial 419 kb 20p13 microdeletion involving the *CSNK2A1* gene, which may represent a potential contributor to an OCNDS-compatible phenotype. In addition to rarely reported features such as intrauterine growth restriction or vesicoureteral reflux, there are pronounced psychiatric abnormalities, including a severe depressive episode with psychotic symptoms that has not previously been described in connection with Okur-Chung neurodevelopmental syndrome.

**Conclusion:**

Assuming that the *CSNK2A1* deletion represents the potential contributor to the OCNDS-compatible phenotype, this case may expand the phenotypic spectrum of OCNDS and highlights the need for a better understanding of the underlying genetic mechanisms and their contribution to the clinical phenotype.

**Supplementary Information:**

The online version contains supplementary material available at https://doi.org/10.1186/s13256-026-06800-7.

## Introduction:

Okur-Chung neurodevelopmental syndrome (OCNDS) (OMIM #617062) is a neurodevelopmental syndrome first described in 2016 [1]. In most cases, it is caused by de novo variants; in rare cases, the disease is inherited in an autosomal dominant manner [1–3]. The disease is caused by changes in the casein kinase II subunit alpha gene (*CSNK2A1* gene), which codes for the alpha subunit of the protein kinase CK2 [4]. The gene is located on chromosome 20 (gene locus 20p13.33) [5]. Current research is focusing on how the known variants in *CSNK2A1* are disease causing; the current assumption is a loss of function, altered substrate specificity and dominant negative mechanisms depending on the variant (particularly the K198R variant) [6, 7]. The protein kinase CK2 is a serine/threonine kinase that is ubiquitously expressed [8]. As early as 2003, over 300 different substrates of this kinase were known. [9] The *CSNK2A1* gene was characterized in more detail in 1998 [10]. The human *CSNK2A1* gene is approximately 70 kb long and contains a total of 14 exons in its primary transcript, with 12 coding exons (UCSC Genome Browser, 2026). The gene regulates the expression of more than 500 downstream genes [11]. Among others, this regulates cell division and differentiation processes [12]. At present, around 100 different disease-causing variants in the *CSNK2A1* gene are known (CSNK2A1 Foundation, 2026). In most cases, the diagnosis is made by Whole Exome Sequencing. For example, 11 cases have been discovered as part of the Deciphering Developmental Disorders Study [8]. The prevalence of the disorder is unknown due to the currently low number of cases. To date, there are around 350 reported patients (CSNK2A1 Foundation, 2026). To date, there seems to be no gender preference (CSNK2A1 Foundation, 2026).

Microdeletions involving the 20p13 region, including the *CSNK2A1* gene, have been reported previously but appear to be rare. Only eleven cases have been described in the literature so far [13–17]. The reported cases vary considerably in both the deletion size and clinical manifestations. However, the underlying pathogenic mechanism and the gene responsible for the associated phenotype have not yet been established. As *CSNK2A1* is consistently affected across the reported cases, its haploinsufficiency has been suggested as a potential contributor to the phenotype [13].

The most common symptoms of OCNDS, which are detectable in almost all patients, include developmental delays and intellectual disability [18]. Common symptoms also include muscular hypotonia, behavioral abnormalities, facial dysmorphia of various types, seizures, as well as feeding disorders. In addition, a number of other symptoms have been described that could be part of the clinical presentation. The exact correlations between genotype and phenotype are the subject of current research due to the small number of patients and the large number of different symptoms. To add new findings about the phenotypic spectrum, we describe the case of a 17-year-old adolescent with an interstitial 20p13 microdeletion encompassing the *CSNK2A1* gene.

## Case report

We report on a 17-year-old male who was treated in our department of child and adolescent psychiatry and psychotherapy. He was the second of three children of non-consanguineous parents to be delivered by primary caesarean section at 37 + 0 weeks gestation. Intrauterine growth retardation due to placental insufficiency had previously been diagnosed: He presented with low birth weight (2480 g, 3rd percentile, −1,95 Standard Deviation [SD] according to World Health Organization Growth Standards and Growth Reference 2007 charts), very short stature (45 cm, <1 st percentile, −2,58 SD) and a normal head circumference (32.5 cm, 6th percentile, −1,54 SD). The amniotic fluid showed a greenish discoloration at birth. The umbilical cord pH at birth was 7.33, the Apgar score after 5/10 min was 8/9. Postnatally, however, short-term intensive medical monitoring was necessary on the first day of life due to a respiratory adaptation disorder. There were initial abnormalities in the hearing screening, but these were not confirmed in a further examination. However, hearing loss was noticed in the subsequent screening examinations—reactions to acoustic stimuli were only recognizable at around 9 months of age. Immediately after birth, the child was in a postural deformity due to a vertebral blockage. Plagiocephaly developed around the age of three months and was treated with helmet therapy. After seven months, he developed microcephaly, which still persists (latest head circumference: 53.2 cm; 2nd percentiles, −1,97 SD, according to KIGGS) [19]. During the first years of life, there was an increased susceptibility to infections, particularly upper respiratory tract infections and urinary tract infections. To the best of our knowledge, immunodeficiency was not investigated. At the age of about one year, ascending pyelonephritis led to inpatient treatment, during which a micturating cystourethrography (MCU) revealed a second degree right vesicoureteral reflux and antibiotic prophylaxis with cefaclor was established. When the urinary tract infection recurred despite prophylaxis, a cystoscopic deflux injection was performed on both sides with a slightly lateralized ostium on the right. The preoperative MCU now showed a second to third degree vesicoureteral reflux on the right and first degree on the left side. Due to recurrent middle ear infections with tympanic effusions, an adeno- and tonsillotomy was performed at the age of 5, followed by multiple paracenteses and tympanic drainage inserts. An electroencephalogram examination at the age of 9 years revealed short (max. 2.5 s), intermittent, generalized, irregular spike-wave paroxysms during sleep. The findings were no longer detectable without seizure suppressant medication and the patient remained completely seizure-free. At the age of 9.5 years, a genetic counseling and detailed examination was carried out. The clinical examination revealed retrognathia, brachydactyly, clinodactyly of digiti V on both sides and the onset of pubic hair with infantile genitalia. Locomotor abnormalities included continued muscular hypotonia, hyperlordosis in the lumbar spine and slight hyperextensibility of the joints. Due to the premature pubarche, as well as a strong weight gain during this period, an endocrinological diagnosis was carried out, which resulted in unremarkable findings. A microdeletion syndrome 22q11 (DiGeorge syndrome) was subsequently ruled out and the chromosome set was numerically unremarkable. An array-based comparative genomic hybridization (Array-CGH) revealed a heterozygous interstitial deletion on the short arm of chromosome 20 (p13), as shown in Table 1.

**Table 1:** Overview of the disease-causing deletion

Chromosomal region	Size	ISCN^a^	RefSeq-genes^b^ involved
20p13	419 kb	arr[GRCh37]20p13(373123_792457) × 1	TRIB3, RBCK1, TBC1D20, **CSNK2A1**, TCF15, SRXN1, SCRT2, SLC52A3

^a^International System for Human Cytogenomic Nomenclature 2016

^b^Reference Sequence genes, Locus Reference Genomic Project of National Library of Medicine

**Fig. 1:**
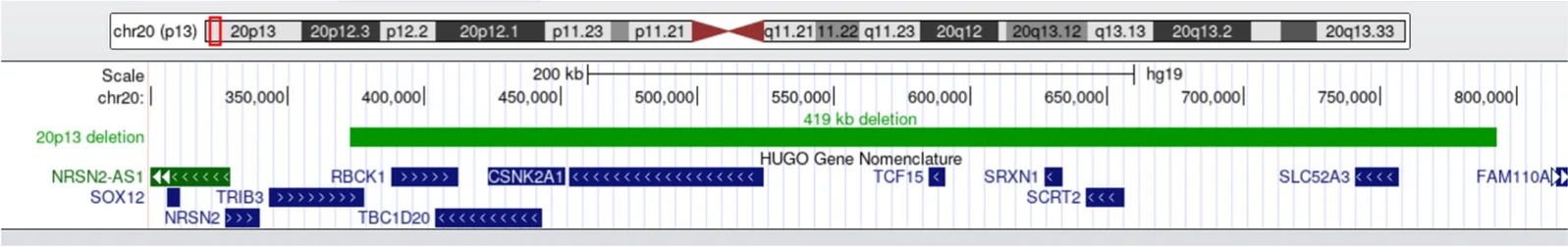
Schematic representation of the 20p13 microdeletion identified in the patient. The deletion is indicated by the green bar and encompasses eight genes, including CSNK2A1. The genomic region and gene positions were visualized using the UCSC Genome Browser (UCSC Genome Browser, 2026)

The interstitial deletion encompasses the entire *CSNK2A1* gene (OMIM #115440, genomic coordinates (GRCh37): 20:453,142–524,434) as well as seven additional genes as shown in Fig. 1. The deletion was not detected in either parent and was therefore presumed to have occurred de novo. No further genetic testing, such as a gene panel for developmental delay, was performed, as the identified deletion was considered the likely underlying cause of the patient’s phenotype.

Also, various developmental abnormalities became apparent in early childhood: On the one hand, an expressive language development disorder (first words spoken at the age of 3 years, short sentences at around 4.5 years), but a gross and fine motor development delay was also described. Primary enuresis nocturna was present until the age of 8. In the first year of school, attention problems, learning difficulties, impulsivity, slight hyperactivity and stereotypical movements that appeared to be mannerisms were noted. The abnormalities mentioned were evident both in the neuropediatric examination and in the results of standardized questionnaires (Child Behavior Checklist (CBCL/4–18) and Teacher Report Form (TRF). At the age of 10, he underwent an assessment for autism spectrum disorder using the Autism Diagnostic Observation Schedule, Second Edition (ADOS-2). The total score of 2 was below the respective ADOS-2 classification threshold. Based on the overall clinical assessment, an autism spectrum disorder was not diagnosed. Various intelligence tests (Kaufman-Assessment Battery for Children II at the age of 7 years; Wechsler-Intelligence Scale for Children – Fifth Edition (WISC-V) at the age of 10 and 17 years) showed a reduction in intelligence (most recently in the borderline below average/mild intellectual disability).

At the age of 17, the patient required child and adolescent psychiatric treatment at our clinic due to acute suicidal thoughts as well as acoustic and visual hallucinations. After the diagnostic evaluation was completed—including the use of psychological self-assessment questionnaires (Beck-Depressions-Inventar Revision (BDI-II), PHOKI (German-language adaptation of the “Fear Survey Schedule for Children – Revised (FSSC-R)”) and Junior Temperament und Charakter Inventar (JTCI 12–18 R)) a severe depressive episode with psychotic symptoms and acute suicidal tendencies was diagnosed. An overview of the key psychological testing performed can be found in supplementary Table 1. During treatment, monotone speech and hypomimia were noted, which according to the child's parents had been present since childhood. The psychotic symptoms remitted almost completely under the administration of the atypical antipsychotic quetiapine (100 mg). No further psychological testing was conducted prior to discharge. An MRI examination of the brain showed unremarkable findings.

## Discussion

Heterozygous loss-of-function variants in the *CSNK2A1* gene are considered to be causative for OCNDS [18]. According to current knowledge, missense variants, which cause a change in the amino acid sequence, are the most common disease-causing variants [20]. There are also null-variants, which seem to lead to a milder phenotype in comparison to missense-variants [21]. In most cases, the variants occur de novo; in rare cases, an autosomal dominant inheritance mechanism has also been described [1–3]. The Array-CGH result arr[GRCh37]20p13(373123_792457) × 1 defines the deleted interval, whereas the CSNK2A1 gene locus (GRCh37: 20:453,142–524,434) lies within this interval. The two coordinate sets therefore describe different genomic features: the deletion span versus the gene locus. The 419 kb interstitial deletion identified in our patient encompasses eight genes: *TRIB3, RBCK1, TBC1D20, CSNK2A1, TCF15, SRXN1, SCRT2* and *SLC52A3*. According to the OMIM database, only three of the eight genes involved, besides *CSNK2A1*, are currently associated with established Mendelian disorders: *RBCK1*, associated with polyglucosan body myopathy 1 with or without immunodeficiency (OMIM #615895); *TBC1D20*, associated with Warburg micro syndrome 4 (OMIM #615663); and *SLC52A3*, associated with Brown-Vialetto-Van Laere syndrome 1 (OMIM #211530). All three disorders are inherited in an autosomal recessive manner, making a pathogenic contribution of a heterozygous loss of a single copy less likely in the absence of a pathogenic variant affecting the remaining allele. In contrast, *CSNK2A1* represents a potential candidate for contributing to the patient’s phenotype, as heterozygous loss-of-function variants are associated with the autosomal dominant disorder OCNDS. In addition, *CSNK2A1* shows a considerable intolerance to loss-of-function variation, with a probability of loss-of-function intolerance (pLI) of 1.0 and a Loss-of-function Observed/Expected Upper Bound Fraction (LOEUF) of 0.27 according to Genome Aggregation Database (gnomAD) and a DECIPHER haploinsufficiency index (HI) of 2.89%. However, the current ClinGen dosage-sensitivity curation considers the evidence for *CSNK2A1* haploinsufficiency to be limited. Among the previously reported cases of 20p13 microdeletions, *CSNK2A1* is included in the deleted region in 11 patients across five publications [13–17]. In one of those cases, Liu et al. proposed *CSNK2A1* haploinsufficiency as a potential mechanism underlying the neurodevelopmental phenotype associated with a 1.59 Mb 20p13 microdeletion [13]. Our patient represents a substantially smaller 20p13 deletion, narrowing the candidate region and further supporting *CSNK2A1* as a potential contributor to the neurodevelopmental phenotype. Nevertheless, a synergistic or modifying effect of haploinsufficiency of the other seven deleted genes cannot be excluded. Taken together, the available evidence suggests that the heterozygous loss of one copy of *CSNK2A1* in the context of 20p13 microdeletion could be consistent with an OCNDS-compatible phenotype, while a contribution of the other deleted genes cannot be ruled out with certainty.

The following section discusses specific phenotypic aspects of the patient case described in comparison to phenotypic aspects of OCNDS.

Some of the symptoms described in this case have rarely been reported in patients with OCNDS: The intrauterine growth retardation found in our patient is described as rare in the most comprehensive case overview to date [18]. Microcephaly is present in about one third of published cases. It is often not apparent at birth, but only appears in the course of early childhood [2]. We could also observe such a development in the patient as shown in Table 2.

**Table 2:** Development of head circumference (HC) within the first two years

Age	HC (cm)	Standard deviation	Percentiles^a,b^
1 day	32.5	− 1.54	6th
5 weeks	35	− 0.74	23rd
15 weeks	38.5	− 1.51	7th
33 weeks	41	− 2.38	1st
13 months	43	− 2.56	1st
23 months	45	− 2.31	1st

^a^According to World Health Organization Growth Standards and Growth Reference 2007 charts

^b^Data adapted to premature birth

A recent study was able to show a correlation between the presence of microcephaly, which also affected our patient, and the localization of the variant: Microcephaly occurred significantly more frequently when the kinase domain of *CSNK2A1* gene was affected [2]. In a review, only about one-third of the patients show abnormalities of the brain on imaging [22]. Recent brain imaging of our patient showed no abnormalities, and in particular, no evidence of any other cause for the psychotic symptoms. Genitourinary abnormalities have rarely been described and are found in this case [8]. Our patient showed brachy- and clinodactyly, which are currently also described as rare [8]. Clinically, he showed impaired hearing in the first year of life. This was not investigated further due to subsequent normalization. Changes in hearing are not described in the review from 2022 [18]. Whether these can nevertheless occur in OCNDS cannot currently be ruled out with certainty.

In addition to a range of primarily physical symptoms, our patient exhibited a range of psychiatric symptoms, along with developmental abnormalities and intellectual disability. These symptoms led to considerable limitations in socio-emotional and educational development in the described case.

Developmental delay and intellectual disability were found in all patients listed in the review article by Okur and Chung from 2022. The language development is described as the most affected domain [18]. Our patient showed reduced intelligence in various intelligence tests administered between the ages of 7 to 16 years, ranging from mild to moderate. The latest test result (WISC-V at the age of 16 years and 8 months) showed an IQ of 69 with especially scoring the "language comprehension" subcategory below average. In terms of developmental delay, the patient showed a delay in language development as well as fine and gross motor developmental delays, which is why he received speech therapy, occupational therapy and physiotherapy in childhood. A review from 2022 reported a prevalence of 19 out of 31 patients for behavioral issues, a category that, in addition to autism spectrum disorder features, also included anxiety and phonic and motor tics in this study [22]. The review article by Okur and Chung reported behavioral abnormalities more frequently in patients with OCNDS (total of 27 out of 36 patients). In detail, stereotypic movements, autism spectrum disorder and attention-deficit/hyperactivity are reported to occur at a rate of approximately 1/3 to 1/5 [18]. Our patient exhibited each of these abnormalities. Attention-deficit/hyperactivity disorder was present in a pronounced form, while psychological testing failed to identify autism spectrum disorder, even though symptoms consistent with autism spectrum disorder were present. Our patient also experienced a severe depressive episode with psychotic symptoms during adolescence. To the best of our knowledge, this has not yet been described as an additional symptom in patients with OCNDS. The oldest patient with OCNDS reported in the literature to date is the only one listed as having depression (though without additional psychotic symptoms) [23]. While severe depressive episode with psychotic symptoms has not been previously detailed in OCNDS, it remains unresolved whether this presentation represents a direct phenotypic expansion of *CSNK2A1* loss, a multigenic contiguous gene effect, or a non-specific psychiatric comorbidity secondary to the severe psychosocial stress of living with a chronic neurodevelopmental disorder. The intellectual disability that is often present in OCNDS, as well as the developmental delays may also predispose for the development of other psychiatric disorders.

The case described illustrates that a 20p13 microdeletion involving *CSNK2A1* may be associated with distinct neuropsychiatric abnormalities. Although the patient does not have a molecularly confirmed diagnosis of OCNDS, the *CSNK2A1* deletion may represent a potential contributor to the OCNDS-compatible phenotype observed in this case. Thus, the present case may expand the phenotypic spectrum associated with heterozygous loss of *CSNK2A1* and highlights the importance of considering neuropsychiatric manifestations in patients with similar genetic findings. Further studies are needed to better understand the contribution of *CSNK2A1* and the other genes encompassed by the 20p13 microdeletion presented in our patient to the observed phenotype, since it remains unclear whether and to what extent the genetic alteration contributed specifically to the neuropsychiatric phenotype. Clinical follow-up should also consider the emergence of neuropsychiatric manifestations during later development, given that neurodevelopmental disorders associated with a 20p13 microdeletion are usually diagnosed in childhood.

## Supplementary Information

Supplementary Material 1.

## Data Availability

No datasets were generated or analysed during the current study.

## References

[1] Okur V, Cho MT, Henderson L, *et al*. De novo mutations in CSNK2A1 are associated with neurodevelopmental abnormalities and dysmorphic features. Hum Genet. 2016;135(7):699–705. 10.1007/s00439-016-1661-y 27048600

[2] Ramadesikan S, Showpnil IA, Marhabaie M, *et al*. Expanding the phenotypic spectrum of CSNK2A1-associated Okur-Chung neurodevelopmental syndrome. Hum Genet Genom Adv. 2025;6(1): 100379. 10.1016/j.xhgg.2024.100379 PMC11621934 39497417

[3] Belnap N, Price-Smith A, Ramsey K, *et al*. Inherited CSNK2A1 variants in families with Okur-Chung neurodevelopmental syndrome. Clin Genet. 2025;104(5):607–9. 10.1111/cge.14408 37491870

[4] Chiu ATG, Pei SLC, Mak CCY, *et al*. Okur-Chung neurodevelopmental syndrome: eight additional cases with implications on phenotype and genotype expansion. Clin Genet. 2018;93(4):880–90. 10.1111/cge.13196 29240241

[5] Blanc A, Bonnet C, Wandzel M, *et al*. Patient with a heterozygous pathogenic variant in CSNK2A1 gene: a new case to update the Okur-Chung neurodevelopmental syndrome. Am J Med Genet A. 2024;194(9): e63642. 10.1002/ajmg.a.63642 38711237

[6] Rushing GV, Sills J. Patient organization perspective: a research roadmap for Okur-Chung Neurodevelopmental Syndrome. Therap Adv Rare Dis. 2024;5:263300402412497. 10.1177/26330040241249763 PMC11273705 39070093

[7] Caefer DM, Phan NQ, Liddle JC, *et al*. The Okur-Chung Neurodevelopmental Syndrome Mutation CK2K198R leads to a rewiring of kinase specificity. Front Mol Biosci. 2022;9:850661. 10.3389/fmolb.2022.850661 PMC9062000 35517865

[8] Owen CI, Bowden R, Parker MJ, *et al*. Extending the phenotype associated with the CSNK2A1-related Okur-Chung syndrome-A clinical study of 11 individuals. Am J Med Genet A. 2018;176(5):1108–14. 10.1002/ajmg.a.38610 29383814

[9] Meggio F, Pinna LA. One-thousand-and-one substrates of protein kinase CK2? FASEB J. 2003;17(3):349–68. 10.1096/fj.02-0473rev 12631575

[10] Wirkner U, Voss H, Ansorge W, Pyerin W. Genomic organization and promoter identification of the human protein kinase CK2 catalytic subunit alpha (CSNK2A1). Genomics. 1998;48(1):71–8. 10.1006/geno.1997.5136 9503018

[11] Borgo C, D’Amore C, Sarno S, Salvi M, Ruzzene M. Protein kinase CK2: a potential therapeutic target for diverse human diseases. Signal Transduct Target Ther. 2021;6(1):183. 10.1038/s41392-021-00567-7 PMC8126563 33994545

[12] Bertrand L, Sayed MF, Pei XY, *et al*. Structure of the regulatory subunit of CK2 in the presence of a p21WAF1 peptide demonstrates flexibility of the acidic loop. Acta Crystallogr Sect D. 2004;60(Pt 10):1698–704. 10.1107/S0907444904016750 15388915

[13] Liu J, Li Y, Andersson HC, Upadia J. Subtelomeric microdeletion in chromosome 20p13 associated with short stature. Clin Case Rep. 2024;12(6): e8927. 10.1002/ccr3.8927 PMC11164670 38863865

[14] Fang HH, Liu SY, Wang YF, Chiang CM, Liu CC, Lin CM. Phenotypic features of a microdeletion in chromosome band 20p13: A case report and review of the literature. Mol Genet Genomic Med. 2019;7(7): e00739. 10.1002/mgg3.739 PMC6625104 31087544

[15] McGill AK, Pastore MT, Herman GE, Alliman S, Rosenfeld JA, Weaver DD. A tale of two deletions: a report of two novel 20p13 –> pter deletions. Am J Med Genet A. 2010;152(4):1000–7. 10.1002/ajmg.a.33339 20358616

[16] An Y, Amr SS, Torres A, *et al*. SOX12 and NRSN2 are candidate genes for 20p13 subtelomeric deletions associated with developmental delay. Am J Med Genet B Neuropsychiatr Genet. 2013;162(8):832–40. 10.1002/ajmg.b.32187 24019301

[17] Jezela-Stanek A, Kucharczyk M, Pelc M, Gutkowska A, Krajewska-Walasek M. 1.15 Mb microdeletion in chromosome band 20p13 associated with moderate developmental delay-additional case and data’s review. Am J Med Genet A. 2013;161(1):172–8. 10.1002/ajmg.a.35654 23165892

[18] Chung W and Okur V. Okur-Chung Neurodevelopmental Syndrome. In: Adam MP, Feldman J, Mirzaa GM, Pagon RA, Wallace SE, Amemiya A, editors. GeneReviews®. University of Washington, Seattle; 2022: 1993–2026. 35679446

[19] Stolzenberg H, Kahl H, Bergmann KE. Körpermaße bei Kindern und Jugendlichen in Deutschland [in German: Body meadurements of children and adolescents in Germany]. Bundesgesundheitsblatt. 2007;50:659–69. 10.1007/s00103-007-0227-5 17514450

[20] Wafik M, Kuoppamaa H, Hirani P, *et al*. Two novel CSNK2A1 variants associated with mild Okur-Chung neurodevelopmental syndrome phenotype. Clin Dysmorphol. 2023;32(3):116–23. 10.1097/MCD.0000000000000456 37195306

[21] Nan H, Chu M, Zhang J, Jiang D, Wang Y, Wu L. Okur-Chung neurodevelopmental syndrome: implications for phenotype and genotype expansion. Mol Genet Genomic Med. 2024;12(3): e2398. 10.1002/mgg3.2398 PMC10915366 38444259

[22] Khamirani HJ, Zoghi S, Motealleh A, *et al*. Clinical features of Okur-Chung neurodevelopmental syndrome: case report and literature review. Mol Syndromol. 2022;13:381–8. 10.1159/000522353 PMC9801326 36588763

[23] O’Donnell S, Goel H. Inherited loss of function variant in CSNK2A1: the oldest reported cases of Okur-Chung syndrome in a single family. Clin Dysmorphol. 2024;33(3):121–4. 10.1097/MCD.0000000000000502 38818820

[24] CSNK2A1 Foundation. 2026. https://www.csnk2a1foundation.org. Accessed 5 May 2026.

[25] UCSC Genome Browser. 2026. https://genome.ucsc.edu. Accessed 20 September 2026.

