# Supplementary material for: Severe neuropsychiatric manifestations in a 17-year-old male with a 20p13 interstitial deletion encompassing CSNK2A1: a case report
Source: J Med Case Rep. 2026 Nov 2;20:675. doi: 10.1186/s13256-026-06800-7 (PMC13729681; doi:10.1186/s13256-026-06800-7)
Supplement: Supplementary file 1 [file 13256_2026_6800_MOESM1_ESM.docx]

| Age of testing^a^ | Psychological testing instrument | Result | Interpretation |
| --- | --- | --- | --- |
| 10;3 | Autism Diagnostic Observation Schedule 2 (ADOS-2)^b^ | 2 points | below the ADOS-2 classification cut-off for ASD (cut-off for autism spectrum disorder according to module one: 11) |
| 10;8 | Wechsler-Intelligence Scale for Children – Fifth Edition (WISC-V)^c^ | IQ: 75 | below-average intellectual functioning (Mean: 100, standard deviation: 15) |
| 16;8 | Wechsler-Intelligence Scale for Children –  Fifth Edition (WISC-V)^c^ | IQ: 69 | approximately two standard deviations below the population mean (Mean: 100, standard deviation: 15) |
| 16;8 | Beck-Depressions-Inventar Revision  (BDI-II)^d^ | 36 points | indicating severe depressive symptom severity (BDI-II range: 29–63) |

*Supplementary Table 1: Overview of the key psychological testing conducted on our patient.*

^a^Age in years; months

^b^ Medda JE, Cholemkery H, Freitag CM. Sensitivity and Specificity of the ADOS-2 Algorithm in a Large German Sample. J Autism Dev Disord. 2019; 49(2): 750-761

^c^ Canivez GL, Grieder S, Buenger A. Construct Validity of the German Wechsler Intelligence Scale for Children-Fifth Edition: Exploratory and Confirmatory Factor Analyses of the 15 Primary and Secondary Subtests. Assessment. 2021; 28(2): 327-352

^d^ Kühner C, Bürger C, Keller F et al. Reliabilität und Validität des revidierten Beck-Depressionsinventars (BDI-II). Befunde aus deutschsprachigen Stichproben [Reliability and validity of the Revised Beck Depression Inventory (BDI-II). Results from German samples]. Nervenarzt. 2007; 78(6): 651-6
